# Persistent unexplained chest pain and dyspnea in a patient with coronary artery disease: a case report

**DOI:** 10.1186/s12872-020-01348-w

**Published:** 2020-02-04

**Authors:** Sameh K. Mobarek

**Affiliations:** North Carolina Heart and Vascular Specialists, UNC/Rex Healthcare, 1505 SW Cary Parkway, Suite 300, Cary, NC 27511 USA

**Keywords:** Autonomic nervous system, Cardiac catheterization, Droxidopa, Neurogenic orthostatic hypotension, Parkinson disease

## Abstract

**Background:**

Neurogenic orthostatic hypotension, a sustained decrease in blood pressure upon standing, is caused by autonomic nervous system failure and characterized by an insufficient increase in heart rate needed to maintain blood pressure upon standing. In this case, neurogenic orthostatic hypotension symptoms preceded a diagnosis of Parkinson disease. A diagnosis of underlying neurogenic orthostatic hypotension significantly changed the course of treatment for this patient.

**Case presentation:**

An 84-year-old woman was referred to a cardiologist by her primary care practitioner for evaluation of exertional dyspnea and chest pain upon walking a few feet. Her medical history included hypertension, hypothyroidism, and osteoarthritis. Based on her continued symptoms, the patient underwent 2 cardiac catheterizations for coronary artery stenosis. After the catheterizations, exertional dyspnea and chest pain continued, and subsequently, dysphagia to solid foods and episodic dizziness developed. Orthostatic evaluation showed a supine blood pressure of 150/80 mmHg with a heart rate of 70 beats per min. Upon standing for 3 min, the patient’s blood pressure decreased to 110/74 mmHg with a heart rate of 76 beats per min. The diagnostic criteria for orthostatic hypotension were met, and the lack of an adequate compensatory heart rate increase upon standing was consistent with a neurogenic cause (ie, neurogenic orthostatic hypotension), which was supported by tilt-table testing results. Although nonpharmacologic treatments were initially successful, episodes of lightheadedness, chest pain, and dyspnea upon standing became more frequent, and the patient was prescribed droxidopa (200 mg; 3 times daily). Droxidopa significantly improved her symptoms, with the patient reporting resolution of her chest pain and significant improvement of dyspnea and dizziness. She was diagnosed with Parkinson disease approximately 6 months later.

**Conclusions:**

This case highlights the importance of evaluating and identifying potential causes of symptoms of cardiovascular disease when persistent symptoms do not improve after cardiac interventions. This case complements findings demonstrating that signs of autonomic failure, such as neurogenic orthostatic hypotension, may precede the motor symptoms of Parkinson disease. Importantly, this case provides real-world evidence for the efficacy of droxidopa to treat the symptoms of neurogenic orthostatic hypotension, after an appropriate diagnosis.

## Background

Orthostatic hypotension is a sustained drop in blood pressure (a drop of ≥20 mmHg systolic or ≥ 10 mmHg diastolic) experienced within a few minutes of standing [1]. Patients with orthostatic hypotension can present with a variety of signs and symptoms, including lightheadedness/dizziness, syncope, and falls [2]. Neurogenic orthostatic hypotension, a subtype of orthostatic hypotension caused by autonomic nervous system failure, can be clinically distinguished from non-neurogenic orthostatic hypotension through autonomic testing and is characterized by the absence of a sufficient compensatory increase in heart rate to maintain blood pressure upon standing [3, 4]. The risk of developing neurogenic orthostatic hypotension increases in patients with neurodegenerative conditions associated with autonomic failure, including Parkinson disease, pure autonomic failure, multiple system atrophy, and dementia with Lewy bodies [5].

Neurogenic orthostatic hypotension often coexists with cardiovascular conditions. Of particular note, approximately 30% of patients with neurogenic orthostatic hypotension have concomitant hypertension, and approximately 50% have concomitant supine hypertension [6, 7]. This constellation of cardiac and orthostatic symptoms can result in multiple referrals and consultations with a variety of primary care practitioners and specialists before a diagnosis of neurogenic orthostatic hypotension is made [8]. This may be especially true in patients without a prior autonomic dysfunction diagnosis (eg, Parkinson disease). In this report, we describe a case of persistent neurogenic orthostatic hypotension symptoms in a patient who underwent cardiac catheterizations followed by percutaneous coronary intervention but was eventually diagnosed with Parkinson disease.

## Case presentation

An 84-year-old woman (5′6″; 144 lb) was referred to a cardiologist by her primary care practitioner for evaluation of exertional dyspnea and chest pain upon walking a few feet. Her medical history included hypertension, hypothyroidism, and osteoarthritis. Her medications included nebivolol, levothyroxine, aspirin, and citalopram. Cardiolite stress test revealed no ischemia, and a 2-dimensional echocardiogram demonstrated normal left ventricular systolic function with no wall motion abnormalities, no significant valvular heart disease, and no pericardial effusion. An electrocardiogram showed normal sinus rhythm, and no acute changes were noted.

Based on her history of continued symptoms, the patient underwent 2 cardiac catheterizations. The first catheterization revealed normal right-sided heart pressures and 80% stenosis of the left circumflex artery and was followed by percutaneous coronary intervention. The second catheterization occurred 2 months after the first and revealed a patent left circumflex artery stent, but 70% stenosis of the right coronary artery, for which the patient again received percutaneous coronary intervention.

The patient continued to experience exertional dyspnea and chest pain, and she subsequently developed dysphagia to solid foods and episodic dizziness. Consultations with a pulmonologist, gastroenterologist, and otolaryngologist revealed no underlying cause for these symptoms apart from evidence of esophageal dysmotility. Her dysphagia persisted despite treatment with a proton pump inhibitor and a histamine H2 receptor blocker. A cardiac event monitor showed no evidence of significant bradycardia or tachycardia, although the patient reported 1 episode of low heart rate (35 beats per min).

**Fig. 1:**
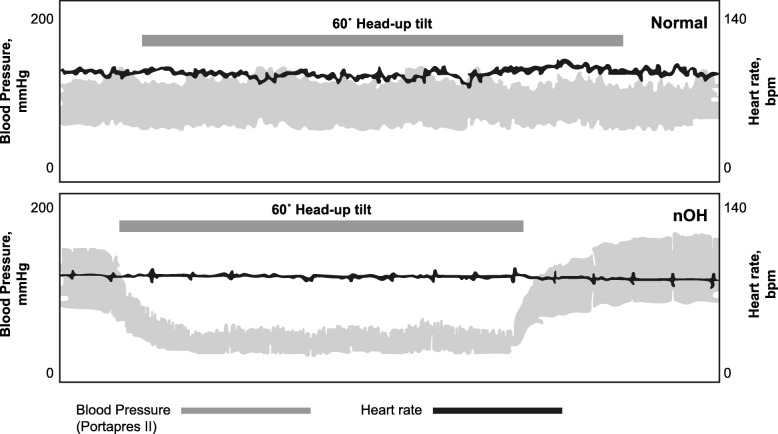
Change in heart rate and blood pressure. Heart rate and blood pressure changes upon 60° head-up tilt in a normal individual (top panel) and in a patient with neurogenic orthostatic hypotension (autonomic failure due to pure autonomic failure; bottom panel) [9]. Normally, heart rate increases upon standing to counteract the effects of gravity on blood pressure, while in neurogenic orthostatic hypotension, there is little or no increase in heart rate to compensate for the decrease in blood pressure upon standing. bpm = beats per minute; nOH = neurogenic orthostatic hypotension. Adapted and republished with the permission of *Clinical Medicine* from Mathias CJ. To stand on one’s own legs. *Clin Med* 2002;2:237–245; permission conveyed through Copyright Clearance Center, Inc.

She was subsequently referred to an electrophysiologist for evaluation of presumed bradycardia. Orthostatic evaluation found a supine blood pressure of 150/80 mmHg with a heart rate of 70 beats per min. Upon standing for 3 min, the patient’s blood pressure dropped to 110/74 mmHg with a heart rate of 76 beats per min. The magnitude of the drop in blood pressure met the diagnostic criteria for orthostatic hypotension (ie, a drop of 20 mmHg systolic or 10 mmHg diastolic), and the lack of an adequate compensatory heart rate increase upon standing was consistent with a neurogenic cause [1, 3, 4]. Tilt-table testing results were also consistent with a diagnosis of neurogenic orthostatic hypotension. The relationship between heart rate and blood pressure upon 60° head-up tilt in a normal individual versus an orthostatic response in a patient with neurogenic orthostatic hypotension is depicted in Fig. 1 [9].

Initially, nonpharmacologic treatments were successful. These included high fluid and increased salt intake; small, frequent, low carbohydrate meals; the use of waist-high compression stockings; aerobic exercises targeted to the lower body; and elevation of the head of the bed. However, episodes of lightheadedness, chest pain, and dyspnea upon standing became more frequent over time. The patient was prescribed droxidopa (200 mg; 3 times daily) for treatment of her symptomatic neurogenic orthostatic hypotension. Droxidopa treatment significantly improved her symptoms, with the patient reporting resolution of her chest pain and significant improvement of dyspnea and dizziness. Six months later, the patient reported gait instability, and upon leaving the exam room, a shuffling gait and unequal arm swings were observed. She was referred to a neurologist and was diagnosed with Parkinson disease, for which she was prescribed carbidopa/levodopa. The patient continued to be followed through the collaborative care of both electrophysiology and neurology.

## Discussion and conclusions

Although patients at risk for neurogenic orthostatic hypotension can present in a variety of clinical settings, cardiologists, in particular may frequently encounter patients with orthostatic symptoms (eg, dizziness/lightheadedness upon standing) and can play an essential role in the recognition and diagnosis of neurogenic orthostatic hypotension. However, referral of such patients to a neurologist to test for other autonomic symptoms, which may provide evidence for an underlying neurodegenerative disease, is recommended. When evaluating patients with persistent orthostatic symptoms of unknown cause, it is important to screen for neurogenic orthostatic hypotension. This screening can be conducted relatively quickly in most clinical settings by asking questions about orthostatic symptoms and taking orthostatic blood pressure and heart rate measurements [2]. Notably, the screening process can be conducted by a trained allied healthcare provider (eg, a nurse) before consultation with the treating cardiologist.

In this patient, symptoms of neurogenic orthostatic hypotension predated motor symptoms and the diagnosis of Parkinson disease by > 6 months. The appearance of neurogenic orthostatic hypotension symptoms in this patient before motor symptoms (ie, shuffling and gait instability) of Parkinson disease is consistent with a case report that demonstrated cardiac sympathetic denervation 4 years before the diagnosis of mild Parkinson disease [10]. Patients who should be screened for neurogenic orthostatic hypotension are therefore not limited to those with known autonomic dysfunction (eg, neurodegenerative conditions, human immunodeficiency virus, diabetes, amyloidosis), but should also include patients who have experienced unexplained falls or syncope, those who are ≥70 years of age and frail or on multiple medications, and those who have orthostatic dizziness or other symptoms that occur only when standing [2].

Formal diagnosis of neurogenic orthostatic hypotension can result in drastically improved symptom management and may obviate the need for further cardiac interventions [8]. A medication review and dose adjustments, if possible, are strongly recommended in patients with neurogenic orthostatic hypotension to help rule out non-neurogenic causes of orthostatic hypotension and to possibly improve patients’ symptoms [2]. Once a diagnosis has been made, nonpharmacologic treatments can be initiated [2], including compression garments, adequate hydration (≥64 oz. per day), increased salt intake, and avoidance of increased body temperature, among other behavioral and dietary interventions [2]. Patients with supine hypertension should elevate the head of the bed and avoid resting in the supine position. If nonpharmacologic interventions do not adequately improve symptoms, concomitant initiation of pharmacologic treatment may be appropriate [2].

Droxidopa is a norepinephrine prodrug approved by the US Food and Drug Administration for treatment of orthostatic dizziness, lightheadedness, or the “feeling that you are about to black out” in adult patients with symptomatic neurogenic orthostatic hypotension caused by primary autonomic failure (Parkinson disease, multiple system atrophy, and pure autonomic failure), dopamine beta-hydroxylase deficiency, and non-diabetic autonomic neuropathy [11]. Droxidopa has been shown in clinical trials to significantly reduce symptoms of neurogenic orthostatic hypotension and the effect of these symptoms on daily activities [12]. Overall, droxidopa has a generally good tolerability and cardiovascular safety profile [12, 13]. Commonly reported adverse events in patients treated with droxidopa during clinical trials were headache, dizziness, nausea, and hypertension [12]. In the clinical trials, the rates of supine hypertension (defined as systolic blood pressure > 180 mmHg) observed in droxidopa-treated patients were low, but were slightly increased compared with placebo (≤7.9% vs ≤4.6%, respectively) [11, 12]. Nevertheless, because patients with neurogenic orthostatic hypotension may also be at risk for concomitant supine hypertension as a consequence of baroreflex failure (ie, regardless of any treatment with pressor agents), the risk of supine hypertension must be carefully managed [2]. In the current case, treatment with droxidopa significantly improved dizziness and dyspnea, with a resolution of chest pain.

This case highlights the importance of evaluating and identifying other potential causes of symptoms that are consistent with cardiovascular disease when faced with persistent symptoms that do not improve with cardiac interventions. Additionally, this case complements findings demonstrating that signs of autonomic failure, such as neurogenic orthostatic hypotension, may precede the motor symptoms of Parkinson disease. Importantly, this case provides real-world evidence for the efficacy of droxidopa to treat the symptoms of neurogenic orthostatic hypotension, after an appropriate diagnosis.

## Data Availability

All data generated or analyzed during this study are included in this published article.
